# Exploration of animal models to study the life cycle of *Fasciola hepatica*

**DOI:** 10.1017/S0031182022000609

**Published:** 2022-09

**Authors:** Wen Fang, Jin Yang, Hai-yin Wang, Shao-rong Chen, Ke-rong Li, Yu-hua Liu, Yi Duan, Tian-mei Li

**Affiliations:** 1 Dali Prefectural Institute of Research and Control on Schistosomiasis, Dali 671000, Yunnan, China; 2 Clinical Medical College, Dali University, Dali 671000, Yunnan, China

**Keywords:** Animal model, *Fasciola hepatica*, Kunming mice, life cycle, rabbit, SD rat

## Abstract

The parasite *Fasciola hepatica* is an important zoonotic parasite. The development of an animal model of *F. hepatica*'s life cycle is critical for studying the biological characteristics of the parasite in snails and mammals. Eggs of *F. hepatica* of bovine origin were cultured, and metacercariae were obtained after infection of *Galba pervia* snails. The life cycle system of *F. hepatica* was initiated in 2 different animals by orally infecting rabbits, SD rats and Kunming mice with the metacercariae. The animals' survival after infection, parasite migration in the animals and pathological damage to the liver were observed. We discovered that rabbits died due to acute suppurative hepatitis 60*–*69 days after infection, and eggs were found in the feces on day 63 of infection. The liver of SD rats showed punctate lesions on day 3 of infection, and further changes occurred as the infection progressed. However, liver repair was observed at week 9. SD rats survived for more than a year after infection and continued the *F. hepatica* life cycle. The liver lesions in Kunming mice after infection were similar but more severe than those in SD rats. Death was observed on the 31st post*-*infection day. We discovered that while rabbits, SD rats and Kunming mice can all be used as animal models of *F. hepatica*, SD rats are more suitable experimental animals in terms of tolerance and pathological response.

## Graphical abstract

**Figure:**
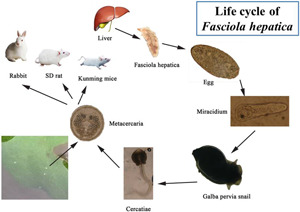

## Introduction

*Fasciola hepatica* causes fascioliasis, a zoonotic foodborne parasitic disease (Wu, 2005). The adult worm of *F. hepatica* parasitizes animals' livers and bile ducts. The eggs, which enter the intestine with bile, are eventually mixed with feces and excreted from the body. Eggs develop into miracidia in water and burrow into Lymnaeidae (intermediate host). The snail develops sporocysts, rediae and cercaria, which then (the cercariae) escape and form metacercariae in the outside world. The metacercariae parasite is in the infective stage. Following ingestion by the mammalian host, the metacercariae excyst due to the action of the gastric juices. The resulting newly excysted juvenile stage penetrates the intestinal wall and enters the peritoneal cavity. The flukes then penetrate the liver envelope, enter the liver and migrate through the parenchyma for several weeks before entering the bile ducts, where they develop into adults.

The intermediate host, the Lymnaeidae, provide the necessary shelter for *F. hepatica* larval development and proliferation. In our previously completed *F. hepatica* infection experiments, *Galba pervia* snails were identified as the intermediate host of *F. hepatica* in Dali (Fang *et al*., 2014). *Galba pervia* snails belong to the Galba in the Lymnaeidae. However, the worm has no strict selectivity for the final host, and a variety of mammals, including humans can be used as its final host.

The life cycle of *F. hepatica* has been successfully established in many animals, such as goats, rabbits, rats and mice (Mango *et al*., 1972; Huang *et al*., 2004, 2006; Hussein and Khalifa, 2008). Meanwhile, a recent study has found that Kunming mice can also be used as infection hosts for *Fasciola gigantica*, which is similar to *F. hepatica* and can cause fascioliasis. These animal models are widely used in biological research, drug development and efficacy evaluation of *F. hepatica*. However, no studies have been conducted to directly compare 3 laboratory small animal models, rabbits, mice and rats, to reveal which small animal model is more suitable for laboratory studies. Therefore, this study will establish a laboratory life cycle of *F. hepatica* in Kunming mice, SD rats and rabbits. The survival of different hosts after infection, as well as the pathological damage that results, will be tracked in order to develop a suitable animal model.

## Materials and methods

### Collection of eggs of *F. hepatica*

A beef stall owner in Dali city was contacted, and after finding the *Fasciola* parasite in the beef liver sold, the seller was notified to collect the parasites. Dali city is a *F. hepatica* and *F. gigantica*, as well as heterozygous infection area (Ai *et al*., 2013; Tang *et al*., 2019). As a result, it is necessary to select worms that have the morphological characteristics of *F. hepatica*. That is, the front end of the worm body has obvious shoulders, the back end is in a ‘V’ shape, 2 intestinal branches with blind ends are parallel and reach the back end of the body, the intestinal branch is divided into many side branches on both sides and the outer branches are numerous and long, while the medial branches are few and short (Lin, 1956; Yin, 1995). After polymerase chain reaction testing to identify *F. hepatica*, the uterus was incised and the eggs were removed.

### Experimental animal and infection

*Galba pervia* snails were captured in a field ditch in the mountainous area of Heqing county, Dali prefecture, without fecal pollution. A total of 66 female SD rats, SPF grade, 5 weeks old, weighing 230–250 g; 60 female Kunming mice, SPF grade, 5 weeks old, weighing 28–30 g (Kunming Nuoshun Biological Technology Co., Ltd., Kunming, China) and 8 female rabbits, 12 weeks old, weighing 2.0–2.2 kg participated in the study (Chaowan Breeding Company, Dali, China).

#### Intermediate host infection

*Galba pervia* snails were captured about a week before infection and kept in a clean water basin. After at least 3 days of observation, no cercariae escaped or metacercariae formation was found in a few snails, which were classified as negative snails. *Fasciola hepatica* eggs were sucked into a weighing bottle, half of which was filled with distilled water and incubated in a water bath at 28°C. After 10–11 days, Miracidia hatched. A 24-well cell culture plate was used to quantitatively infect the snails, and the ratio of snails to miracidia was 1:3–1:5. Snails were cultured and metacercariae were collected with methods commonly used in our laboratory (Fang *et al*., 2014). The temperatures of the room and the water were recorded on a daily basis.

### Infection of the final host

#### Rabbit infection

First, the rabbits were checked for infection by parasites. The feces of 8 rabbits were collected for 3 consecutive days using the nylon silk egg collection method (Chen, 1988). The fecal residue was aspirated and examined under a microscope for the presence of parasitic eggs. The rabbits that were not infected with parasites were randomly divided into experimental and control groups. There were 2 rabbits in the control group, both of which were not fed with metacercariae. A total of 6 rabbits was randomly divided into 2 groups of 3 rabbits each (numbered A1–3 and B1–3). Fifteen fresh metacercariae were fed orally to each rabbit in group A and 30 in group B. The rabbits were observed for tolerance to infection and pathological changes in the liver. From the 7th week of infection, rabbit feces were collected daily and examined for liver lamellate trematode eggs in the feces using the nylon silk collection method until eggs were detected.

#### SD rat infection

Rats that were not infected with the parasite were randomly divided into pre-experimental, experimental and control groups, and the experimental group was further randomly divided into 2 groups, experimental group A and experimental group B. Fourteen rats in the pre-experimental group were fed with 30 fresh metacercariae each. Two rats were dissected on alternate days from 1 to 14 days after infection, respectively. The migration of the worms in the rats, the pathological damage to the liver and the detection of the worms were observed. The experimental protocol was adjusted according to the results of the pre-experiment. Thirty-one rats in group A were fed orally with 20 fresh metacercariae each. Two rats were dissected each day from 1 to 8 days after infection, 1–2 rats each week from 11 to 20 weeks and 2 rats each for 6 months and 1 year. Thirteen rats in group B were fed with 30 fresh metacercariae. At 4–10 weeks after infection, 1–2 rats were dissected each week. Eight rats in the control group were not fed with metacercariae. One was dissected on the day of infection and 1 on the 10th post-infection day, and 1 per month thereafter. The migration and development of the worms in the rats were observed. The survival time and the pathological changes in the liver of each group were recorded. The miracidium were also collected from the livers of dissected rats and used to study the life cycle of *F. hepatica* in Kunming mice.

#### Kunming mice infection

The uninfected Kunming mice were randomly divided into experimental and control groups. A total of 51 mice in the experimental group was divided into 3 groups, A, B and C, with 17 mice in each group. Two fresh metacercariae were fed orally to each mouse in group A, 6 in group B and 10 in group C. Two to 3 mice were dissected on the 3rd, 7th, 28th, 49th and 70th days after infection, respectively. Nine mice in the control group were not fed with metacercariae, and 1–2 mice were dissected at the corresponding days. The migration and development of the worms in the mice were observed. The survival time and the pathological changes in the liver of each group were recorded. The tolerance to the different numbers of metacercariae in each group of mice was analysed.

### Drug efficacy observation

Two rabbits that were in a state of dying after being fed 30 metacercariae orally and infected with *F. hepatica* were analysed. To ensure that the worms were completely killed, 2 courses of triclabendazole oral treatment were given. An interval of 2 weeks was allowed between the 2 courses of treatment to adequately observe the effect of the drug. The human oral dose of triclabendazole was 10 mg kg^−1^ (1 time day^−1^) for 2 days. The dose for rabbits was 2.3 times higher than that for humans. The oral drug dose for rabbits was 10 mg × 2.3 × rabbit body weight day^−1^ × 2 days. The rabbits were weighed before and after each course of treatment. And all the feces of each rabbit were collected daily to observe the excretion of worm eggs. The collected eggs were incubated separately in a water bath at 28°C and the development of eggs was observed for 10 consecutive days. Two rabbits were dissected on the 11th day after the second course of treatment.

### Section of pathological tissue

A sample of liver tissue was taken and fixed in a sample preservation solution (formula: 150 mL formaldehyde, 8 g sodium dihydrogen phosphate, 12 g disodium hydrogen phosphate, 1000 mL distilled water) and handed over to the Dali State Hospital Department of Pathology for paraffin sectioning and haematoxylin and eosin staining. Under the microscope, histopathological changes were observed.

### Statistical analysis

Microsoft Excel 2007 was used to enter and organize the experimental data. The survival rates of the 3 animals were compared using SPSS Statistics 26.0 software at a significance level of 0.05.

## Results

### Infection of the snails and metacercariae harvest

In this experiment, 1214 *G. pervia* snails were infected, and 657 snails were positive. The positive rate is 54.1% (657/1214). Until the metacercariae harvest, 203 positive snails remained and 2907 metacercariae were collected from them. At the same time, 148 inactive dead metacercariae were found, accounting for 5.1% (148/2907) of all metacercariae. The dead metacercariae are characterized by normal appearance with an intact 3-layer capsule wall. The metacercariae within the capsule were scattered and inactive after removing the outermost capsule wall. Alternatively, the morphology of the posterior cercaria in the capsule was ambiguous.

### Rabbit infection

**Fig. 1.**
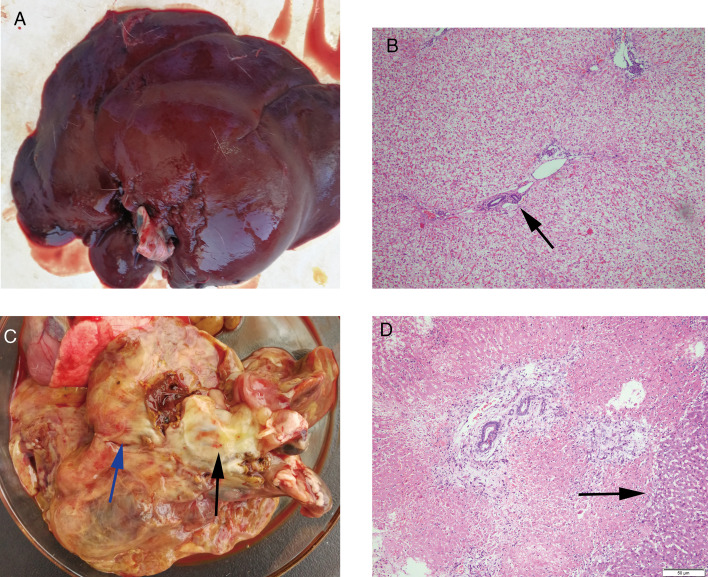
Histological and pathological changes in the liver of rabbits. (A) Livers of rabbits in the control group. (B) Portal area of rabbits in the control group (original magnification: 40×). (C) Pseudomembranes (blue arrow), abscesses (black arrow) and necrosis on the surface of the liver of rabbits infected for 68 days. (D) The liver pathology of rabbits infected for 68 days showed extensive coagulative necrosis (original magnification: 200×). The black and blue arrows indicate the described feature.

On the 63rd day after infection, eggs were discovered in feces. A rabbit died on the 60th day in group B (B2), displaying pathological signs of acute liver inflammation. The cellulose exudates on the surface of the liver formed a thick ‘pseudomembrane’ (Fig. 1C). Cellulose exudates have also been discovered to adhere to the stomach, intestines and other organs. Pathological signs included liver swelling, bleeding, abscesses of various sizes, viscous, bean curd-like pus, gallbladder enlargement and blackening. A total of 9 worms with sizes of 1.0–2.0 × 0.2–0.7 cm^2^ were isolated, 5 from the intrahepatic bile duct, 2 from the common bile duct and 2 from the gallbladder. Group A rabbits 1–3 died on the 67th, 68th and 69th days, respectively. The liver damage caused by A1 and A3 rabbits was comparable to that caused by B2. The liver of A2 rabbit, on the other hand, was found to be yellowish-grey, with local haemorrhage, hard, inelastic, inconspicuous pseudomembrane and a large amount of pleural and ascites. Following liver incision, several purulent foci with white pus were obtained. A histopathological section of the liver was taken, which revealed extensive coagulative necrosis (Fig. 1D). Three mature adult worms measuring 2.0–2.7 × 0.8–0.9 cm^2^ were extracted from the bile duct. The rabbits in the control group were dissected at 60 and 97 days, and no abnormalities were found.

### SD rat infection

**Fig. 2.**
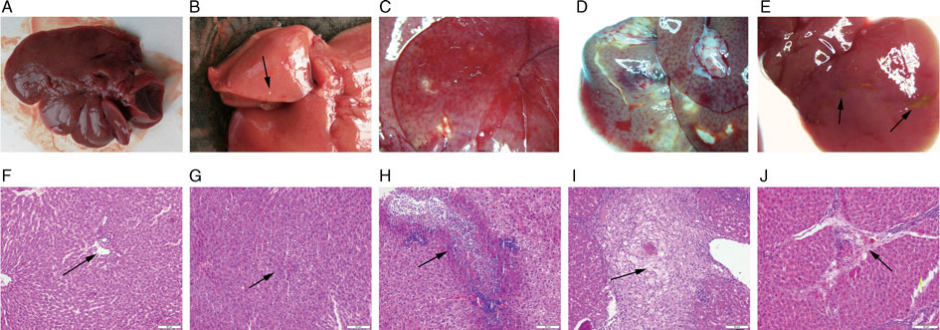
Histological and pathological changes in the liver of SD rats. (A) Livers of rats in the control group. (F) Portal area of rats in the control group (original magnification: 40×). (B) On the 3rd day after infection in rats, punctate lesions appeared on the surface of the liver. (G) Liver pathology in rats infected for 3 days showed focal necrosis (original magnification: 200×). (C) After 7 days of infection in rats, linear lesions appeared on the surface of the liver. (H) Seven days after infection, eosinophilic abscesses appeared in the liver pathology of rats (original magnification: 40×). (D) Abscesses, haematomas and abscess tracts appeared on the surface of the liver of rats infected for 4 weeks. (I) Liver pathology in rats infected for 4 weeks showed foam cell aggregates (original magnification: 200×). (E) A few necrotic tissues remained unrepaired on the surface of the liver of rats infected for 1 year. (J) Pathology of the liver of rats infected for 1 year showed tissue regeneration and repair (original magnification: 40×). The black arrow indicates the described feature.

On the 3rd day after infection in rats, milky white punctate lesions were seen in the liver (Fig. 2B), and focal necrosis was seen in pathological sections (Fig. 2G), indicating that *F. hepatica* had reached the liver through the intestine and abdominal cavity, which is the earliest. On the 3rd day of infection, the parasites could reach the liver and cause pathological damage to the liver. As a result, it began to migrate and disrupt the liver on a continuous basis, forming dot-shaped, linear and sheet-shaped ‘worm passages’ along its path. The ‘worm duct’ was initially congested and exuded inflammatory fluid, which gradually transformed into haematoma and abscess. Multiple linear lesions appeared on the 7th day (Fig. 2C). Pathology showed eosinophilic abscess of the liver with extensive tissue necrosis (Fig. 2H). On the 12th day after infection, the first worm body (size: 0.133 × 0.055 cm^2^) was discovered in the liver. At 4 weeks, the abscess on the liver expanded and fused, and necrosis also appeared around the haematoma, accompanied by bloody ascites and peritoneal adhesions (Fig. 2D), and the pathological section showed the accumulation of foam cells in the liver tissue (Fig. 2I) and the worm was 0.4–0.6 × 0.12–0.3 cm^2^. The migration of the worms to the bile duct was documented 5 weeks after infection; the worms were immature and measured to be 0.7–0.9 × 0.2–0.3 cm^2^. At 9 weeks, the liver pathology showed a state of repair, the damaged part was shrunk due to repair and the liver and the gastrointestinal tract were adhered, the size of the worm was 1.0–2.0 × 0.5–0.9 cm^2^ and some of the eggs were mature. At 19 weeks, further restoration of the damaged liver tissue was reduced and concentrated on the injured part in the 1–2 lobes. The mature worm measured 3.0 × 1.2 cm^2^ in length. After 6 months to a year of infection, the liver was reddish-brown with a smooth surface and a few linear or small yellow lesions were observed locally (Fig. 2E). Pathological sections showed regeneration and repair of liver tissue (Fig. 2J).

Four rats infected with 20 metacercariae in experimental group A died on the 58th day. Anatomical examination revealed severe liver damage, including extensive necrosis, several abscesses, adhesion with surrounding organs, bloody pleural effusion and ascites. Furthermore, the remaining rats were able to progress in accordance with the experimental design, and 2 rats infected with 20 metacercariae survived for more than a year. The death of 1 of them on the 375th day could be attributed to liver cirrhosis. Simultaneously, the other surviving rat was dissected. The liver is shown in Fig. 2E and J. Rats in the control group had no abnormalities (Fig. 2A and F).

### Infection with 4 Kunming mice

**Fig. 3.**
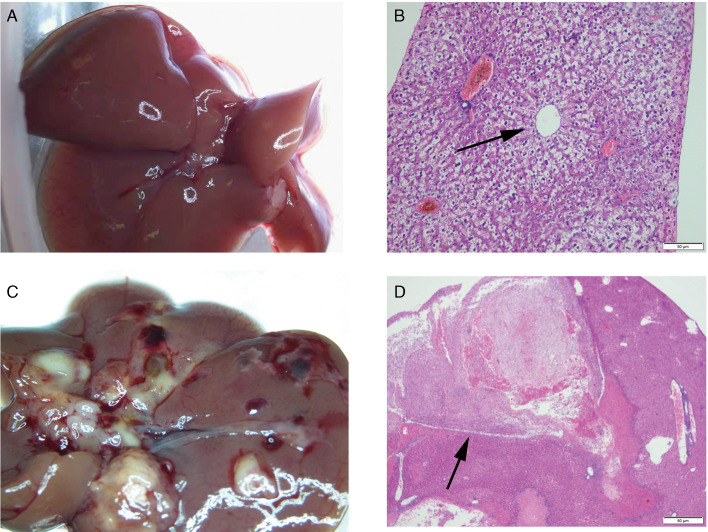
Histological and pathological changes in the liver of Kunming mice. (A) Livers of Kunming mice in the control group. (B) The central vein of the mice liver in the control group (original magnification: 200×). (C) Haematomas and abscesses appeared on the surface of the liver of mice infected for 68 days. (D) Liver pathology in mice infected for 4 weeks showed extensive necrosis (black arrow; original magnification: 40×).

On the 3rd day after infection, Kunming mice fed 2, 6 or 10 metacercariae developed punctate lesions in the liver. On the 7th day, as the number of infected metacercariae increased, the range of lesions on the liver intensified, and the lesions evolved from punctate to linear, as seen in rats. However, after 4 weeks, the liver of mice with different infections revealed pathological manifestations that were nearly identical. They all had the pathological features of acute inflammation: fibrinous exudation, bloody ascites, a liver abscess, a haematoma, a thick pseudomembrane and organ adhesion (Fig. 3C). Pathological sections showed extensive necrosis of liver tissue (Fig. 3D). The mice with varying numbers of metacercariae died one after the other beginning on the 31st day, with the first death occurring in the mice infected with 2 metacercariae. At 34 days, only 1 mouse infected with 10 metacercariae survived. However, it was dissected because it appeared to be on the verge of death. So far, all mice infected with 10 metacercariae have been euthanized.

### Situation of rabbits after drug administration

The effect of oral triclabendazole on 2 dying rabbits, B1 and B3, was observed. From 1 to 6 days after administration, more eggs were consistently detected in the feces of rabbit B3, while rabbit B1 had less feces and fewer or sometimes no eggs were detected. However, all eggs found in the feces of rabbits B1 and B3 showed no signs of development. At the end of the first course of treatment, the body weight of rabbit B1 increased by 0.5 kg compared to the pre-dose period, while that of rabbit B3 increased by 0.6 kg. There was no significant change in body weight at the end of the second course of treatment.

**Fig. 4.**
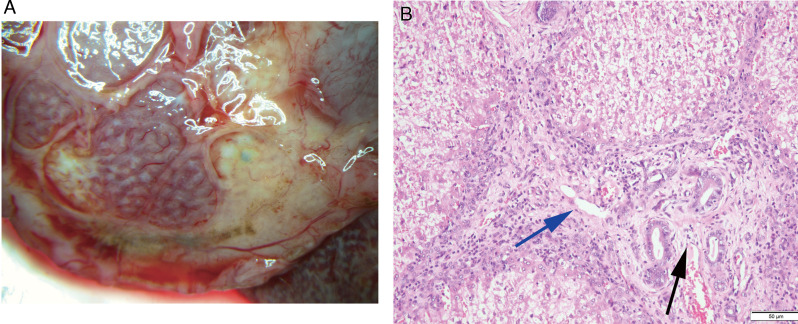
Liver repair in rabbits after 2 treatments. (A) The liver rebuilt itself into ‘liver lobules’. (B) Pathology showed granulation tissue formation in the liver, with visible fibrous tissue (blue arrow) and small bile duct (black arrow) hyperplasia (original magnification: 200×).

On day 11, rabbit B1 was dissected and found to have a thick pseudomembrane on the surface of the liver with adhesions to the surrounding organs. A faintly structured worm was found in the hepatic bile duct. The gallbladder completely adherent to the liver, and the bile in the gallbladder was in the form of streaks with white debris suspected to be worm fragments. The liver had shown obvious repair. Hepatic lobules formed by fibrous tissue hyperplasia were visible to the naked eye (Fig. 4A), and pathological sections showed granulation tissue formation fibrous tissue hyperplasia and bile duct hyperplasia (Fig. 4B). After dissection of rabbit B3, several abscesses were found on the pseudomembranes with the maximum size of 4 × 5 × 3 cm^3^. No worms were found in the liver. Many eggs were found floating in the clear bile. The shell of the eggs was intact and the egg cap was clear, but no oocytes were seen and the yolk cells were mostly in fragments. The pathological presentation of the liver was similar to that of B1.

### Life cycle maintenance

Since *F. hepatica* has completed part of its life history in rats and the viability of rats was not significantly affected 6 months after infection. Therefore, worms from rats infected for 6 months were collected and further used for the collection and incubation of eggs. The miracidia were captured after 10 days of incubation and infected 28 snails. The sporocysts were discovered on the 6th day after infection, the rediae were discovered on the 24th and the metacercariae were harvested on the 39th. In total, 549 metacercariae were collected. There were 42 dead metacercariae, accounting for 7.7% (42/549). To investigate whether the metacercariae collected during this process are infective, the collected metacercariae were used to infect Kunming mice.

### Statistical investigation

The Kruskal*–*Wallis test was used on the survival status of the 3 animal models. The mortality difference between the 3 animal models was statistically significant (*F* *=* 17.065, *P* *<* 0.05). A pairwise comparison was performed on this basis, and the results revealed that the mortality rate of SD rats was lower than that of the other 2 animals (*P* < 0.05), and the difference was statistically significant. The mortality of the 3 animals infected with *F. hepatica* with different numbers of metacercariae is shown in Table 1.

**Table 1.** Mortality of 3 animals infected with *Fasciola hepatica* and recovery of flukes

Species	Numbers infected	Number of fed metacercarial	Number of deaths		Time to detect eggs (days)	Recovery of parasites from host
			No.	%		
Rabbits	3	15	3	100	63	15
	3	30	3	100		9
Rats	31	20	4	12.9	63	20
	13	30	0	0		33
Mice	17	2	3	17.6	49	2
	17	6	5	29.4		9
	17	10	7	41.2		10

The rabbits taking triclabendazole were moribund and thus classified as dead for statistical purposes.

## Discussion

*Fasciola hepatica* is known to be a common parasite capable of infecting herbivores, humans and dozens of other mammals. Moreover, fascioliasis caused by it is still a parasitic disease that poses a serious threat to human health and hinders the economic development of animal husbandry. Currently, research on *F. hepatica* has focused on large herbivores in animal husbandries, such as cattle, sheep and deer. However, humans are also very much affected as occasional hosts of this parasite, especially in rural areas there is higher prevalence of human fascioliasis (Sah *et al*., 2018). Therefore, the study of *F. hepatica* causing human fascioliasis should also receive special attention.

Due to the specificity of the study subject, we were unable to focus on humans directly. And the biological characteristics of large herbivores differ much from those of humans, which significantly limits our further research on human infection with *F. hepatica*. Therefore, it is crucial to find animal models that exemplify the host–parasite interaction mechanism between humans and *F. hepatica*. We chose rabbits, SD rats and Kunming mice as experimental subjects. Rabbits are monogastric herbivores and natural hosts of *F. hepatica*. Although SD rats and Kunming mice are not natural hosts, they have physiological characteristics similar to humans and can also be infected with *F. hepatica*. On the one hand, these 3 types of animals are the most commonly used in biological research. On the other, they are inexpensive and easy to manage during experiments. So, in this study, we screened for more suitable animal models that simulate human conditions by observing the susceptibility of animals to *F. hepatica*, the developmental status of the worm in the body, the pathological damage caused to the organism and the survival. It will help us to lay the foundation for further study of human fascioliasis.

During the process of obtaining metacercariae, we found about 5–8% of dead metacercariae. This has not been observed in previous experiments and has not been reported in other reports. It would be of great benefit if the mechanism of natural death of metacercariae could be further investigated and applied to the control process of *F. hepatica*. Meanwhile, it suggests that the presence of ‘dead metacercariae’ should be excluded when used for animal infection, especially the infected dose is as small as 2–5 metacercariae. Otherwise, there is a possibility of infection failure.

This study showed that all 3 animals were susceptible to *F. hepatica* and that the worms could develop and mature in all 3 animals. This indicates that they can all serve as important infection hosts for *F. hepatica*, which is consistent with the findings of Burden *et al*. (1983). However, we found some characteristics of each of the 3 animals after infection with *F. hepatica*. Comparing these characteristics can more visually indicate that *F. hepatica* has a large variation in virulence and invasiveness in different types of hosts (Cwiklinski *et al*., 2018).

SD rats have the longest survival time among the 3 types of animals, with eggs detectable in the feces after 9 weeks of infection. Meanwhile, *F. hepatica* not only completes a complete life cycle in rats, but also develops into infective worms that can be used to establish a life cycle in other animal models. This is a very important finding that small omnivores can provide developmental sites for *F. hepatica* and that well-developed insects can also lay eggs for subsequent life cycle maintenance. Moreover, SD rats as conserved hosts of *F. hepatica* can provide worms at different developmental stages to study.

The pathological examination of the rat's liver showed that the liver had started to repair itself from the 9th week after infection, such as aggregation of eosinophils and repair of large areas of fibrous tissue. But also, there were many signs of damage features such as necrosis, abscesses and foam cell formation (Frigerio *et al*., 2020). These pathological manifestations are essentially the same as those in humans infected with *F. hepatica* (Nishioka *et al*., 2021). In fact, most patients with human fascioliasis have a long chronic course. Therefore, the pattern of ‘damage and repair’ exhibited by SD rats fits well with that of humans. Moreover, this is more conducive to studying the immune effect mechanism between the parasite and host. It will help scientists to develop new interventions in a more targeted manner.

The growth and development of *F. hepatica* in Kunming mice are very rapid. The length of the worms in mice can attain 2.0–2.5 × 0.5–0.9 cm^2^ *in vivo* in the 7th week after infection. This is in agreement with the results reported by Dawes (1962). It also caused very severe damage to the liver and led to the gradual death of the mice from the 4th week. Meanwhile, we found that the rate and number of deaths in mice increased with the number of infected metacercariae. Kunming mice infected with 10 metacercariae dead in 34 days. Therefore, if mice are used for longer experiments, the number of infected metacercariae should preferably be 1–2 (Dawes, 1962). There was a study reported that Kunming mice can be used as a research model for *F. gigantica*. However, in our experiments, though *F. hepatica* completed a complete life cycle in mice, the mice died quickly. This also suggests that the severity of *F. gigantica* and *F. hepatica* causing the same disease in the same class of animals may be inconsistent (Mei *et al*., 2021). Besides, another limitation is that the amount of blood in mice is small and not easy to collect. Especially in cases where the liver is severely damaged, sometimes it is not even possible to collect blood. But the blood specimens are very important which can be used in many basic studies, such as immunology. Thus, the infection characteristics in Kunming mice suggest that this animal model can be used for studies related to the acute phase of *F. hepatica* infection.

Eggs could also be found in the feces of rabbits 9 weeks after infection, indicating that *F. hepatica* had also completed its life cycle in rabbits. In the next observations, it was found that the rabbits also had very severe liver damage. There was no significant difference in the results of infections with 30 or 15 metacercariae. The effect of both doses on the liver pathology of rabbits was approximately the same, with severe infectious hepatitis. This result is different from the one reported by Huang *et al*. (2004) for rabbits infected with metacercariae of *F. hepatica* of French origin. This may be because we used eggs isolated from cattle liver from the Dali region of Yunnan, China. It also suggests that there may be large differences in virulence between different geographical sources of *F. hepatica*.

In the observation of the efficacy of triclabendazole, we found very instructive phenomena. During the collection of eggs in the feces, triclabendazole appeared to be more effective in rabbit B1 than that in rabbit B3, because the number of eggs in the feces of rabbit B1 was small. However, after we dissected both rabbits, the situation was reversed. In rabbit B1, we found not only broken worms that were not completely excreted, but also a solidification of its bile. This solidified bile could, on the one hand, interfere with the expulsion of worms and eggs. On the other, it may cause very serious diseases, such as biliary tract infection and gallbladder cancer. This situation suggests that it may not be sufficient to observe the presence of eggs in the patient's stool when treating fascioliasis. Medical imaging should also be used to ensure that the treatment is thorough and safe. In addition, we found that the eggs collected after the administration of the drug were not active and could not develop. This also proves that triclabendazole can destroy *F. hepatica*.

Although *in vitro* experiments can also reveal some of the mechanisms, animal models are irreplaceable compared to *in vitro* studies. For example, *in vitro* experiments cannot simulate the prolonged infection state of *F. hepatica*, but they can be fully simulated in animals. In contrast, the study of imageology is only possible in animal models (Han *et al*., 1999).

In conclusion, the results of the extent of pathological damage caused to the host after *F. hepatica* infection and the survival time were analysed. We believe that SD rats are most suitable for the laboratory construction of long-term animal models of *F. hepatica* infection. Also, mice and rabbits can be chosen as models if they are used to study the immunological and pathological changes in animals in the early or acute phase after infection.
